# Study on the protective effect of berberine treatment on sepsis based on gut microbiota and metabolomic analysis

**DOI:** 10.3389/fnut.2022.1049106

**Published:** 2022-12-19

**Authors:** Huibin Pan, Lixia Huo, Weiyun Shen, Zhuquan Dai, Ying Bao, Chaohui Ji, Jie Zhang

**Affiliations:** ^1^Emergency Intensive Care Unit, The First Affiliated Hospital of Huzhou University, The First People's Hospital of Huzhou, Huzhou, Zhejiang, China; ^2^Huzhou Key Laboratory of Translational Medicine, The First Affiliated Hospital of Huzhou University, The First People's Hospital of Huzhou, Huzhou, Zhejiang, China; ^3^Department of Surgery, The First Affiliated Hospital of Huzhou University, The First People's Hospital of Huzhou, Huzhou, Zhejiang, China

**Keywords:** sepsis, berberine, gut microbiota, metabolomics, 16S rDNA

## Abstract

**Introduction:**

Sepsis, an infection with multiorgan dysfunction, is a serious burden on human health. Berberine (BBR), a bioactive component, has a protective effect on sepsis and the effect may be related to gut microbiota. However, studies on the role of BBR with gut microbiota in sepsis are lacking. Therefore, this study investigated the ameliorative effects and the underlying mechanisms of BBR on cecal ligature and puncture (CLP) rats.

**Methods:**

This study has observed the effect of BBR on pathological injury, Inflammation, intestinal barrier function, gut microbiota, and metabolite change in CLP rats by Hematoxylin-eosin staining, enzyme-linked immunosorbent assays, flow cytometry, 16S rDNA, and metabolomics analyses.

**Results:**

The inhibition effects of BBR treatment on the histological damage of the lung, kidney, and ileum, the interleukin (IL)-1b, IL-6, IL-17A, and monocyte chemokine-1 levels in serum in CLP rats were proved. Also, the BBR inhibited the diamine-oxidase and fluorescein isothiocyanate-dextran 40 levels, suggesting it can improve intestinal barrier function disorders. The cluster of differentiation (CD) 4^+^, CD8^+^, and CD25^+^ Forkhead box protein P3 (Foxp3) + T lymphocytes in splenocytes were up-regulated by BBR, while the IL-17A+CD4+ cell level was decreased. The abundance of gut microbiota in CLP rats was significantly different from that of the sham and BBR treatment rats. The significantly changed metabolites in the serum mainly included carbohydrates, phenols, benzoic acids, alcohols, vitamins et al. Additionally, this study predicted that the biological mechanism of BBR to ameliorate sepsis involves glycolysis-, nucleotide-, and amino acid-related metabolic pathways.

**Discussion:**

This study proved the strong correlation between the improvement effect of BBR on sepsis and gut microbiota and analyzed by metabolomics that gut microbiota may improve CLP rats through metabolites, providing a scientific basis for BBR to improve sepsis and a new direction for the study of the biological mechanism.

## 1. Introduction

Sepsis is a prevalent problem among humans of all ages and is a common cause of death in the intensive care unit (1). Modern studies suggest that sepsis is an infection with organ dysfunction, and systemic inflammatory response syndrome is the main clinical manifestation (2). In addition, as the disease progresses, immunosuppression and other symptoms, including organ dysfunction, appear, eventually leading to death from organ failure (3). The regulation of immune homeostasis is key to sepsis treatment. The distribution of deaths in sepsis shows a trimodal pattern, with an increase in deaths during the initial hyperinflammatory phase, weeks of persistent organ dysfunction, and 2–3 months after sepsis, all associated with immune dysregulation (4). In the hyperinflammatory phase, scientists found that various proinflammatory cytokines such as interleukin (IL)-1β, IL-6, monocyte chemoattractant protein-1 (MCP-1), and IL-17A levels were significantly elevated and considered to be one of the inducements of organ dysfunction (5–7). Therefore, the proinflammatory cytokines have become therapeutic targets for sepsis. For example, researchers found that IL-17A has critical effects on host defense, cell trafficking, immune regulation, and tissue repair and played a key role in triggering innate immune defense (8). Although the awareness of sepsis in the scientific community is continuously improving, the overall survival rate of patients is still not optimistic (9). Therefore, the study of the pathogenesis and treatment of sepsis is important and urgent.

As the main bioactive component extracted from *Phellodendron* bark and *Coptis japonica* in traditional Chinese medicine, berberine (BBR) has been widely used to treat patients with abdominal pain, diarrhea, or gastroenteritis with few adverse events (10). It has been proven to have anti-inflammatory, antioxidant, anti-atherosclerotic, antibacterial, anti-tumor, and neuroprotective properties (11, 12). Yan and co-workers found that BBR protected the intestinal mucosal barrier in sepsis by regulating Zrt-Irt-like protein 14 expression and zinc redistribution (13). In addition, BBR can inhibit the levels of MCP-1 and other chemokines in cecal ligature and puncture (CLP)-rat tissues to improve organ damage in combination with yohimbine (14). However, the biological mechanism of BBR in improving sepsis is still insufficient.

Modern studies claimed that the gastrointestinal tract is the largest immune organ in the body and that intestinal barrier interactions, gut microbiota, and immune system cells are at the core of regulating immune homeostasis (15). Studies showed that an imbalance of intestinal microbial homeostasis can cause sepsis susceptibility (16). Additionally, Liu and his co-workers studied clinical stool samples from some patients with sepsis and found that the severity of sepsis was higher in mice that received stool transplants from those patients (17). Furthermore, scientists found that metformin can reduce liver injury in sepsis rats by regulating the gut microbiota (18). It is suggested that improving gut microbiota homeostasis plays a crucial role in reducing sepsis injury. More and more evidence proves that the biological effects of BBR are closely related to gut microbiota (19). A study found that the improvement effect of BBR with a low bioavailability on atherosclerosis is inseparable from its ability to regulate the remodeling of gut microbiota (20).

Similarly, Zhang found that BBR did not directly affect lipopolysaccharide-induced microglial activation *in vitro*; however, BBR could significantly change the gut microbiota of fecal colonized rats from patients with irritable bowel syndrome and inhibit visceral hypersensitivity and microglial activation in rats (21). To the best of our knowledge, studies on the role of BBR and gut microbiota in sepsis are lacking. Therefore, this study investigated the ameliorative effects and the underlying mechanisms of BBR on sepsis rats with CLP-induced sepsis through 16S rDNA and metabolomic analysis, which provided a scientific basis for developing and treating sepsis. It is expected that it will provide a research basis for future research into the biological mechanism of BBR.

## 2. Material and methods

### 2.1. Animals

The Sprague Dawley (SD) rats were purchased from Shanghai Jihui Laboratory Animal Care Co., Ltd. (SCXK (Hu) 2017-0012). All procedures on animals followed the Public Health Service Policy on Humane Care and Use of Laboratory Animals. After 7 days of adaptive feeding, the 200–230 g SD rats were divided into three groups for CLP model construction: the sham group, the CLP group, and the CLP + BBR group. Before the CLP surgery, SD rats were injected intraperitoneally with BBR (berberine hydrochloride, dose: 50 mg/kg; B21449, Yuanye, Shanghai, China) or PBS (as a solvent control) one time a day for 5 days. The CLP rats were anesthetized with isoflurane (R510-22-10, RWD, San Diego, CA, USA) and then cut from the middle of the abdominal wall. The cecum was gently pulled out to ligate at one-third from the end of the cecum with sterile No. 4 thread. Then, a 1 ml injection needle (21 G) was used to puncture the perforation in the middle between the ligature site and the top of the cecum about 3–4 times. The rats were sutured and resuscitated with 5 ml/100 g of normal saline. The sham rats underwent surgery similar to that performed on CLP rats, except for cecal ligation and puncture.

### 2.2. Sample collection

After 6 h of the CLP operation, a sample of blood was drawn from the abdominal aorta of anesthetized SD rats. In brief, the anesthetized rats in the left supine position were exposed to their viscera and moved their viscera gently to expose the spine. Further, there were two blood vessels in front of the spine, of which the right was the abdominal aorta. Then, blood sampling needles and vacuum blood collection tubes (without anticoagulant) were used to collect ~2 ml of blood. Additionally, the blood was centrifuged at 3,000 rpm for 10 min to get the serum. The serum was divided into 0.2 ml in each tube, snap frozen in liquid nitrogen, and stored at −80°C separately (22). Then, PBS perfusion was performed from the right atrial appendage, and the lungs, the kidneys, and the ileum tissues were taken and stored at −80°C.

### 2.3. Histological examination

The histological examination of the lungs, the kidneys, and the ileum was performed as described previously by hematoxylin-eosin (H&E) staining (23). The H&E staining kit was purchased from Wuhan Servicebio Biotechnology Co., Ltd., China. The extent of histological damage in the lungs, the kidneys, and the ileum was evaluated as previously described (24–26). The score was proportional to the extent of the damage. In summary, the score for the lungs was based on the degree of alveolar cavity integrity and inflammatory cell aggregation (24). The score for the kidneys was based on the degree of swelling and shedding of epithelial cells and inflammatory cell aggregation (25), and the score for the ileum was based on the integrity of the lamina propria, capillary congestion, and degree of mucosal separation (26). All evaluations were semi-quantitative scores and were performed by pathologists without grouping information. The experiments, as described previously, were performed by operators who knew nothing about the groups of sections.

### 2.4. Detection of inflammatory cytokines

According to the instructions, the inflammatory cytokines were measured by enzyme-linked immunosorbent assays (ELISA). The IL-6 (MM-0190R2, MEIMIAN, Jiangsu, China), IL-17A (MM-70049R2, MEIMIAN, Jiangsu, China), IL-1β (MM-0040M1, MEIMIAN, Jiangsu, China), and MCP-1 (MM-0099R2, MEIMIAN, Jiangsu, China) were used for evaluating the level of inflammation in serum. The optical density was measured at 450 nm by a microplate reader (CMaxPlus, Molecular Devices, San Jose, CA, USA). Their concentration was directly proportional to the optical density (450 nm) value, which was calculated by plotting a standard curve.

### 2.5. Detection of intestinal barrier function

At 18 h after CLP, rats in each group were gavaged 750 mg/kg of fluorescein isothiocyanate (FITC)-dextran 40 (FD-40; 60842-46-8, Sigma, San Antonio, TX, USA). The superior mesenteric vein blood was obtained, and the serum was separated 6 h after gavage. The absorbance of serum in each group was measured with a Beckman fluorescence spectrophotometer (Indianapolis, Indiana, USA; the excitation light and emission light were 490 and 520 nm, respectively), and the concentration of FD-40 in the superior mesenteric vein blood was calculated based on the standard curve. In addition, the plasma obtained from inferior vena cava blood was used for measuring the activity of diamine oxidase (DAO) according to the DAO ELISA kit (MM0237R1, MEIMIAN, Jiangsu, China) instructions.

### 2.6. Assessment of the immune response degree

The flow cytometry assay was used for the assessment of the immune response degree as described by Pérez-Cano et al. (27). The sterile spleen tissues were used for obtaining cells, and PBS was used for adjusting the concentration of cell suspension to 2 × 10^7^ cells/ml. Subsequently, the BD OptiBuild™ BV480 Mouse Anti-Rat Cluster of Differentiation (CD)8 (746832, BD, USA), the BD Pharmingen™ PE-Cy™7 Mouse Anti-Rat CD4 (561578, BD, Franklin Lakes, USA), the BD Pharmingen™ PE Mouse Anti-Rat CD3 (554833, BD, USA), the Recombinant Rat IL-17A protein (778701, BioLegend, USA), the Forkhead box protein P3 (FOXP3) antibody (sc-65988, Santa Cruz Biotechnology, Dallas, Texas, USA), and BD Pharmingen™ FITC Mouse Anti-Rat CD25 (553072, BD, NJ, USA) were added for incubation in the dark.

### 2.7. 16S rDNA sequencing

After euthanasia, the cecum contents of six rats in each group were taken and handed over to Hangzhou LianChuang Biomedical Tech Co., Ltd., China. The 16S rDNA sequencing was performed as described by Gao's team (28). After the quality evaluation of the total DNA, the genome was amplified by PCR to obtain the V3–V4 region of the bacterial 16S rRNA gene. The PCR amplification was recovered and quantified. According to the sequencing quantity requirements of each sample, each sample was mixed according to the corresponding proportion. Then, high-throughput sequencing was conducted after normalizing the PCR libraries. The sequence de-noising or operational taxonomic units (OTU) clustering was performed according to the Divisive Amplicon Denoising Algorithm (DADA2). Subsequently, Quantitative Insights into Microbial Ecology (QIIME2) was also used for species composition, alpha diversity, and beta diversity analysis. The correlation analysis of gut microbiota abundance was carried out by SparCC (SparCC is a Python module for calculating correlations in compositional data).

### 2.8. Metabolomics analysis

Metabolomics analyses of serum were performed by Metabo-Profile Biotechnology Co. Ltd., Shanghai, China. Briefly, serum sample pretreatment mainly consisted of thawing and centrifugation to separate debris and fat. Then, 175 μl of pre-cooled methanol/chloroform (volume ratio = 3/1) was mixed with a mixture containing 50 μl of sample and 10 μl of internal standard, and these mixtures were centrifuged to get the supernatant. Then, 200 μl supernatant was added into an autosampler vial (Agilent Technologies, Foster City, CA, USA). The remaining sample supernatant was used to prepare quality control samples. After the autosampler vial containing the sample was subjected to a step involving the removal of chloroform and drying, 50 μl methoxyamine (20 mg/ml in pyridine) and 50 μl *N*-methyl-*N*-(trimethylsilyl) trifluoroacetamide (1% trimethylchlorosilane; Thermo-Fisher Scientific, Fairlawn, NJ, USA) containing fatty acid methyl ester (C7–C30, Sigma-Aldrich, Saint Louis, MO, USA) were sequentially added. The processed samples were injected into the analytical equipment. The nontargeted metabolomics approach had been previously described by Zhao et al. (29). The metabolomics analysis performed by the Gas Chromatography-Time-of-Flight Mass Spectrometry (GC-TOF/MS) platform (Gerstel, Germany) integrated processing samples and issuing reports. The iMAP software was used to perform data analysis. Moreover, the correlation analysis of gut microbiota abundance and metabolomics was used in the R (4.2.1) project for statistical computing. ANOVA analysis yielded a *p*-value of 0.05 and |log_2_ (fold change) | 30 based on the ratio of the means.

### 2.9. Statistical analysis

A one-way ANOVA followed by the Tukey test and the *t*-test were employed in the data analysis of measurement data comparing various groups using SPSS 16.0 (IBM, Armonk, NY). The Kruskal–Wallis *H*-test was used for those with uneven variance. All data were expressed as mean ± standard deviation, and a *p*-value < 0.05 indicates that the difference was statistically significant.

## 3. Result

### 3.1. BBR treatment on the CLP rats improved the histological injury of the lungs, the kidneys, and the ileum

**Figure 1:**
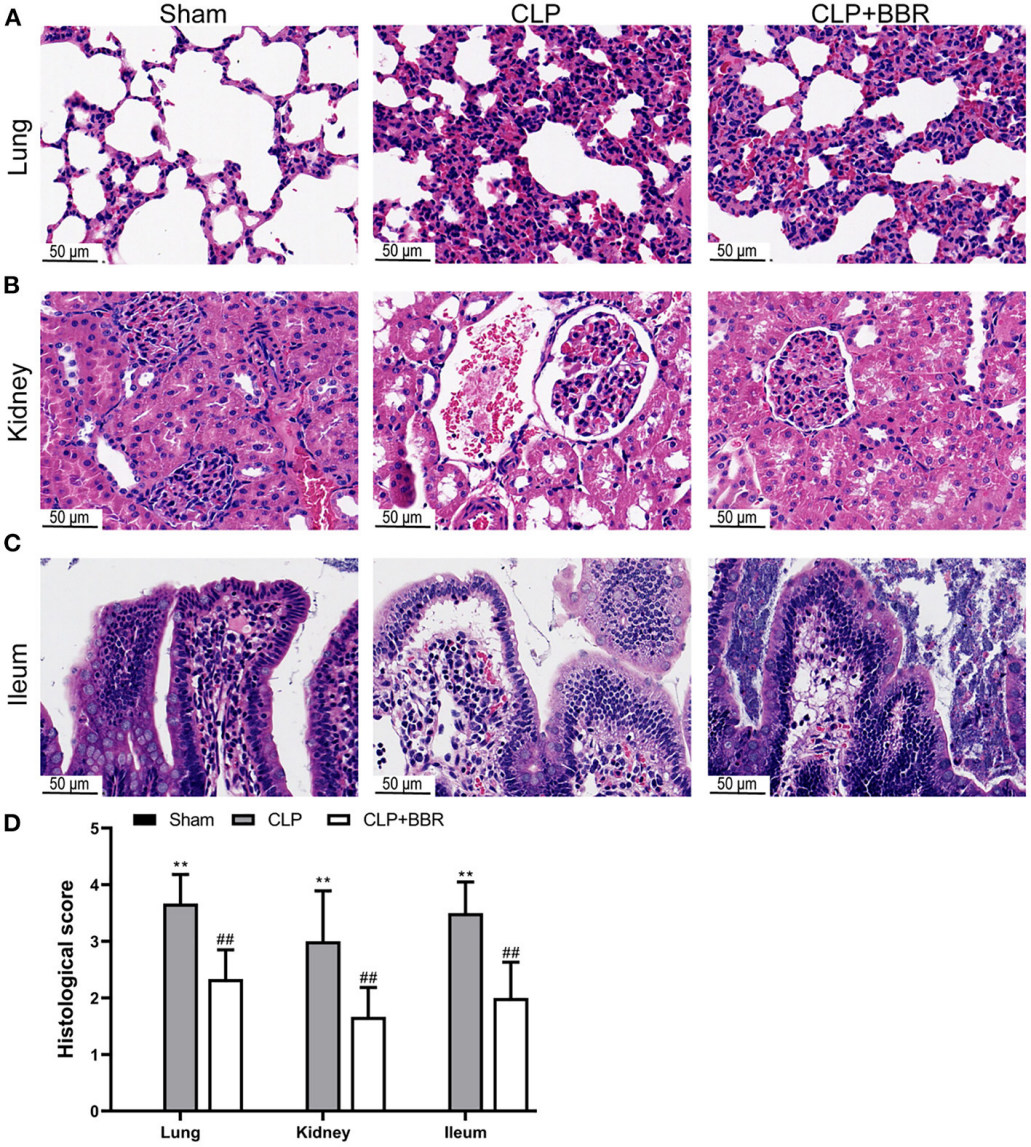
Histological observation of cecal ligature and puncture (CLP) rats by hematoxylin-eosin staining. Berberine treatment on sepsis improved the histological damage of **(A)** lung, **(B)** kidney, and **(C)** ileum, scale bars = 50 μm. Also, berberine treatment on sepsis decreased **(D)** histological scores of the lung, kidney, or ileum (*n* = 6). Data are shown as mean ± standard deviation. **p < 0.01 vs. sham group; ^##^*p* < 0.01 vs. CLP group.

H&E staining detected any histological injury. There were no obvious pathological changes in the sham group's lungs, kidneys, or ileum. Further, the CLP group's lungs were observed with alveolar space narrowing, inflammatory cell infiltration, and alveolar wall thickening (Figure 1A). In the kidney, the sham group's glomerulus was without injury and shrinkage, while the glomerulus of the CLP group showed shrinkage, and there were clear inflammatory cell infiltration and many necrotic and exfoliated epithelial cells in the kidneys (Figure 1B). In addition, in the CLP group, the ileum was observed with severe edema and infiltration of inflammatory cells, accompanied by a large number of necrotic and exfoliated epithelial cells (Figure 1C); healing of all the above injuries improved in the CLP + BBR group. The histological scores also showed that the histological injury of the lungs, the kidneys, and the ileum in CLP rats after BBR treatment was significantly improved (*p* < 0.01; Figure 1D).

### 3.2. BBR treatment on the CLP rats improved the inflammation and gut barrier function

**Figure 2:**
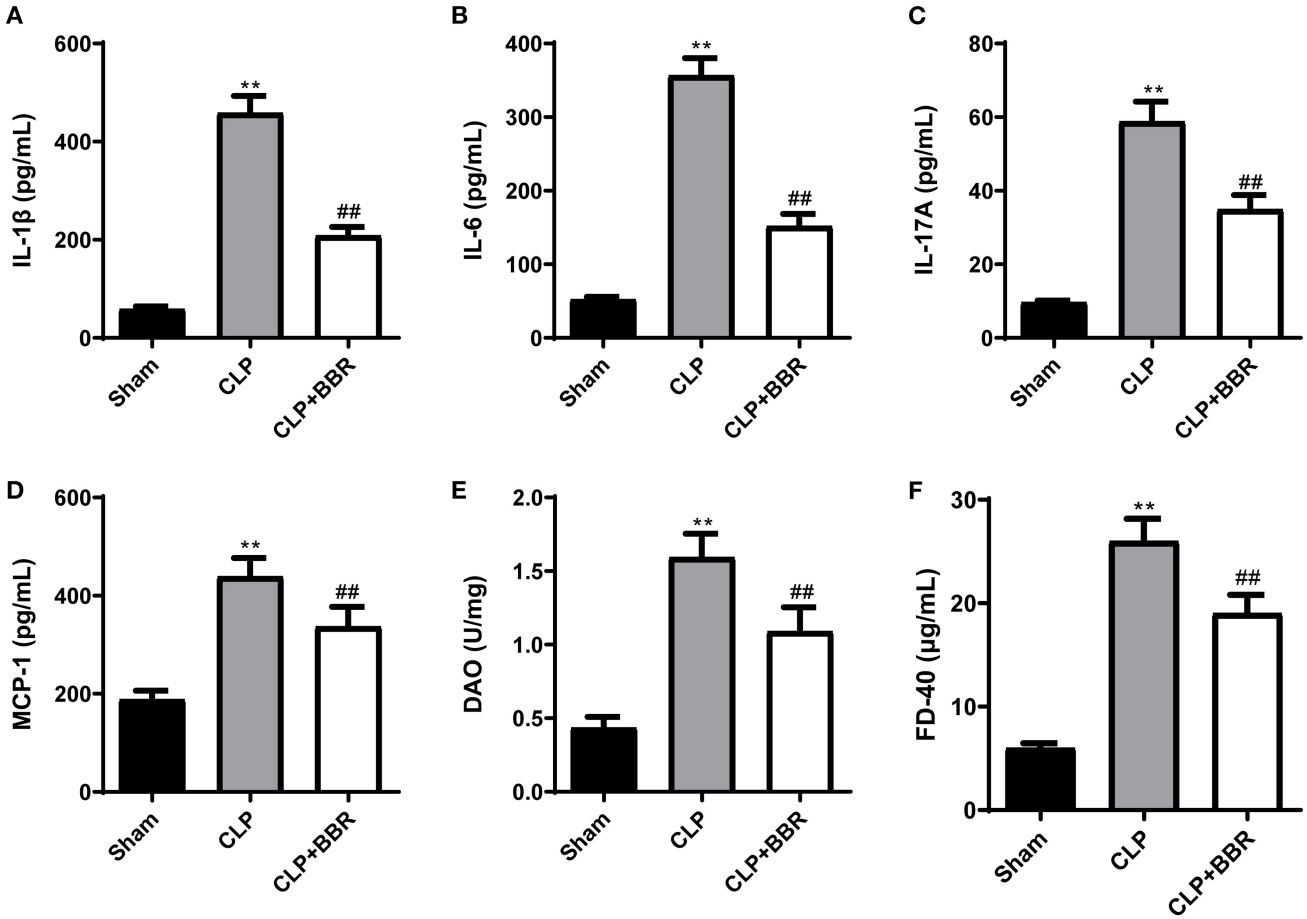
Berberine improved the inflammation and the gun barrier function in cecal ligature and puncture rats. The **(A)** interleukin (IL)-1β, **(B)** IL-6, **(C)** IL-17A, and **(D)** monocyte chemoattractant protein-1 (MCP-1) levels in the serum of CLP rats were decreased by berberine treatment. The **(E)** diamine oxidase (DAO) and **(F)** fluorescein isothiocyanate-Dextran 40 (FD-40) levels in the plasma of CLP rats were used to measure the gut barrier function and berberine treatment decreased them (*n* = 6). Data are shown as mean ± standard deviation. **p < 0.01 vs. sham group; ^##^*p* < 0.01 vs. CLP group. CLP, cecal ligature and puncture; BBR, berberine.

The levels of inflammatory cytokines in serum were detected using ELISA. The levels of IL-1β, IL-6, IL-17A, and MCP-1 were notably increased using CLP (*p* < 0.01), while BBR treatment decreased them (*p* < 0.01; Figures 2A–C). The levels of DAO and FD-40 in the plasma of the CLP group were higher compared to the sham group (*p* < 0.01; Figures 2E,F). BBR treatment markedly downregulated DAO and FD-40 levels (*p* < 0.01).

### 3.3. The regulatory effect of BBR treatment on the percentage of T cells in CLP rats

**Figure 3:**
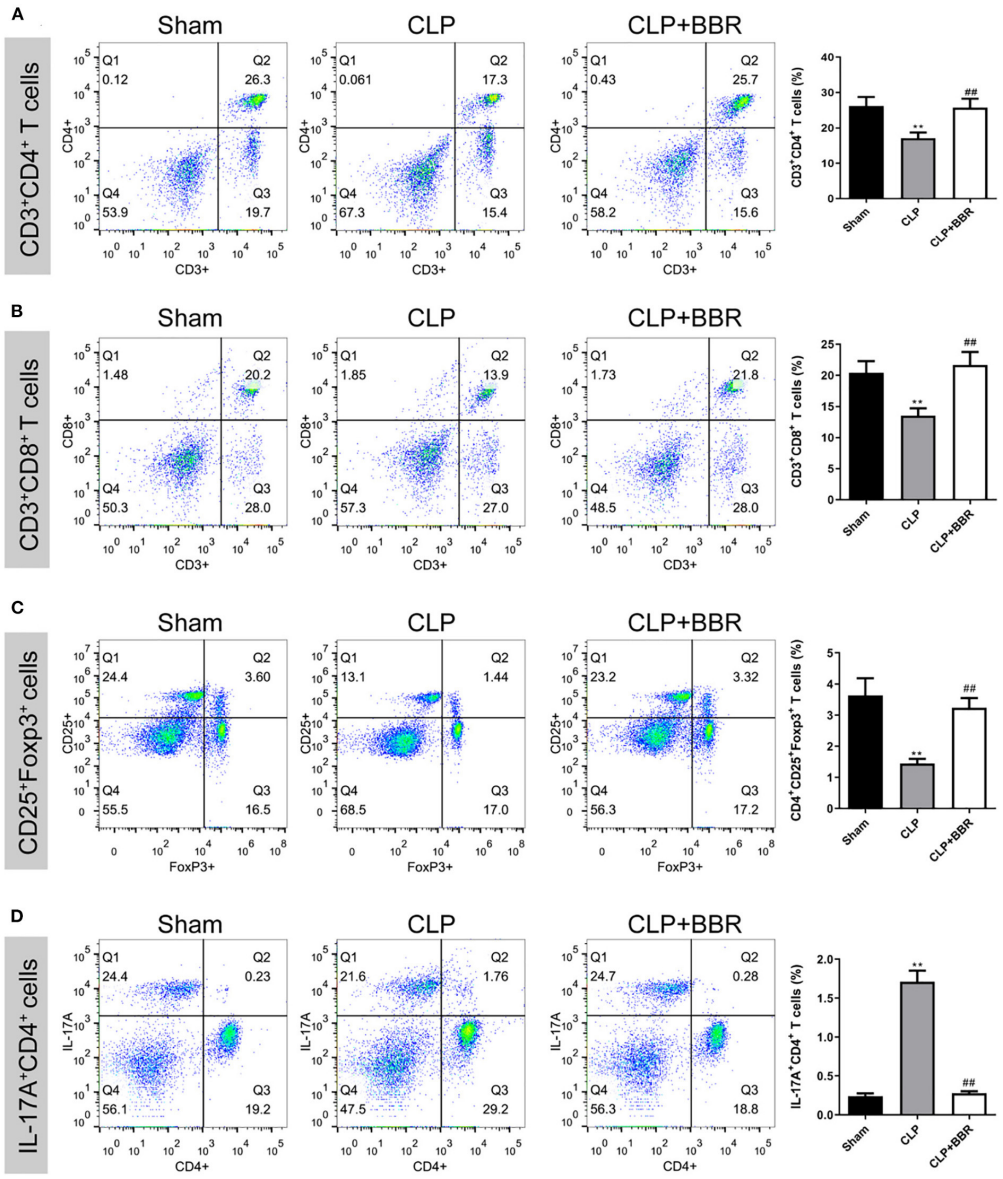
The percentage of CD4^+^, CD8^+^, CD4^+^CD25^+^ Foxp3^+^, and IL-17A^+^CD4^+^ T cells in splenocytes measured by flow cytometry. Berberine treatment increased the proportion of **(A)** CD3^+^CD4^+^, **(B)** CD3^+^CD8^+^, and **(C)** CD4^+^CD25^+^Foxp3^+^ T cells and inhibited **(D)** the IL-17A^+^CD4^+^ T cells in splenocytes of CLP rats (*n* = 3). Data are shown as mean ± standard deviation. **p < 0.01 vs. sham group; ^##^*p* < 0.01 vs. CLP group. CD, cluster of differentiation; Foxp3, forkhead box protein P3; IL, interleukin; CLP, cecal ligature and puncture; BBR, berberine.

The percentage of CD3^+^ CD4^+^, CD3^+^ CD8^+^, CD25^+^ Foxp3^+^, and IL-17A T cells in splenocytes was detected using flow cytometry. As Figure 3 shows, in the CLP group compared to the sham group, the percentages of CD3^+^CD4^+^ (Figure 3A), CD3^+^CD8^+^ (Figure 3B), and CD4^+^CD25^+^Foxp3^+^ (Figure 3C) T cells in spleen cells were notably decreased (*p* < 0.01), especially the percentage of IL-17A^+^CD4^+^ T (Figure 3D) cells, which was remarkably increased (*p* < 0.01). Further, BBR treatment antagonized the changes in the percentage of T cells in CLP rats (*p* < 0.01).

### 3.4. Changes in gut microbiota composition in CLP rats after BBR treatment

**Figure 4:**
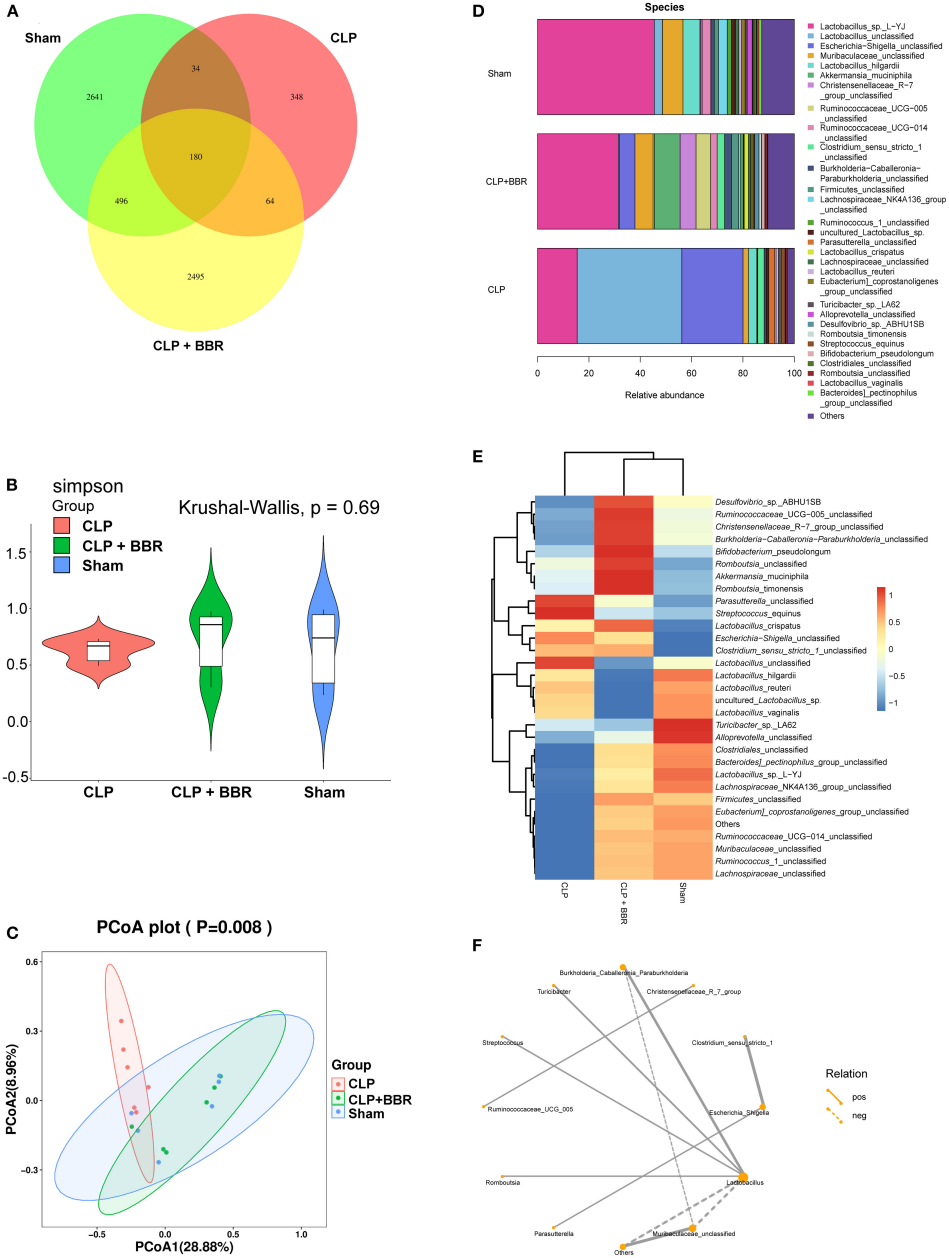
Berberine improved the gut microbiota homeostasis in CLP rats. **(A)** The venn of feature expression abundances. **(B)** The ameliorative effect of berberine on the gut microbial species richness was analyzed by the Simpson index of alpha diversity analysis. **(C)** The principal coordinates analysis (PCoA) was based on the feature abundance table to observe the differences between samples. **(D)** Histogram and **(E)** heat map of the relative abundance of the gut microbiota in Sham, CLP, and CLP + BBR groups (top 30). Heat map was generated by taking the mean values within a biological replicate group, with blue representing lower abundance and red representing higher abundance. **(F)** It is a network of SparCC analysis about the genus-level abundance of the top 30 gut microbiota, which shows the relationship pairs of correlation coefficient |rho| > 0.4. The line is thicker, the correlation is stronger. And the solid line indicates a positive correlation, and the dotted line indicates a negative correlation. The size of the node indicates the number of other bacteria associated with the bacteria. The more associations, the node is larger, on the contrary, the node is smaller.

The 16S rDNA was used for analyzing the gut microbiota of all three groups. According to the eigenvalue abundance table of gut microbiota, there are 180 common features among these three groups, and an additional feature unique to the sham group was 2,640, while 348 features were unique to the CLP group, and 2,495 features were unique to the CLP + BBR group (Figure 4A). In addition, using the Simpson index of alpha diversity analysis, we observed that it was not significant in the three groups (*p* > 0.05; Figure 4B). Moreover, the result of the principal coordinates analysis (PCoA) in beta diversity analysis showed that the abundance of species in the CLP group was different from the sham group and the CLP + BBR group, and the beta diversity analysis of the sham group and the CLP + BBR group was similar (Figure 4C).

The 30 bacteria shown in Figures 4D,E were the top 30 in abundance at the species level among the three groups. They were *Lactobacilus_sp._L-YJ, Lactobacilus_unclassified, Escherichia-Shigella_unclassified, Muribaculaceae_unclassified, and Lactobacillus_hilgardii*, among others. Further analysis of its expression at the species level revealed that the *Lactobacillus_crispatus* was significantly increased in CLP rats compared to the sham rats. On the contrary, the *Lactobacillus_unclassified, Lactobacillus_hilgardii, Lactobacillus_reuteri*, and *Lactobacillus_vaginalis* were significantly decreased in the CLP + BBR group. Conversely, *Muribaculaceae_unclassified* was increased in the CLP + BBR group compared to the CLP group. *Clostridiales*_unclassified was upregulated in the sham group and the CLP + BBR group compared to the CLP group (Figures 4D,E).

As shown in Figure 4F, *Lactobacillus* was the key gut microbiota with the most relevant gut microbiota. Further, *Muribaculaceae_unclassified* and others had a negative correlation with *Lactobacillus*. The *Burkholderia_Caballeronia_Paraburkholferia, Turicibacter, Streptococcus*, and *Romboutsia* had a positive correlation with *Lactobacillus* (Figure 4F). We also observed that *Clostridium_sensu_stricto_1* and *Parasutteralla* had a positive correlation with *Escherichia_Shigella* (Figure 4F). In addition, *Christensenellaceaae_R_7*_group and *Ruminococcaceae_UCG_005* had a positive correlation (Figure 4F).

**Figure 5:**
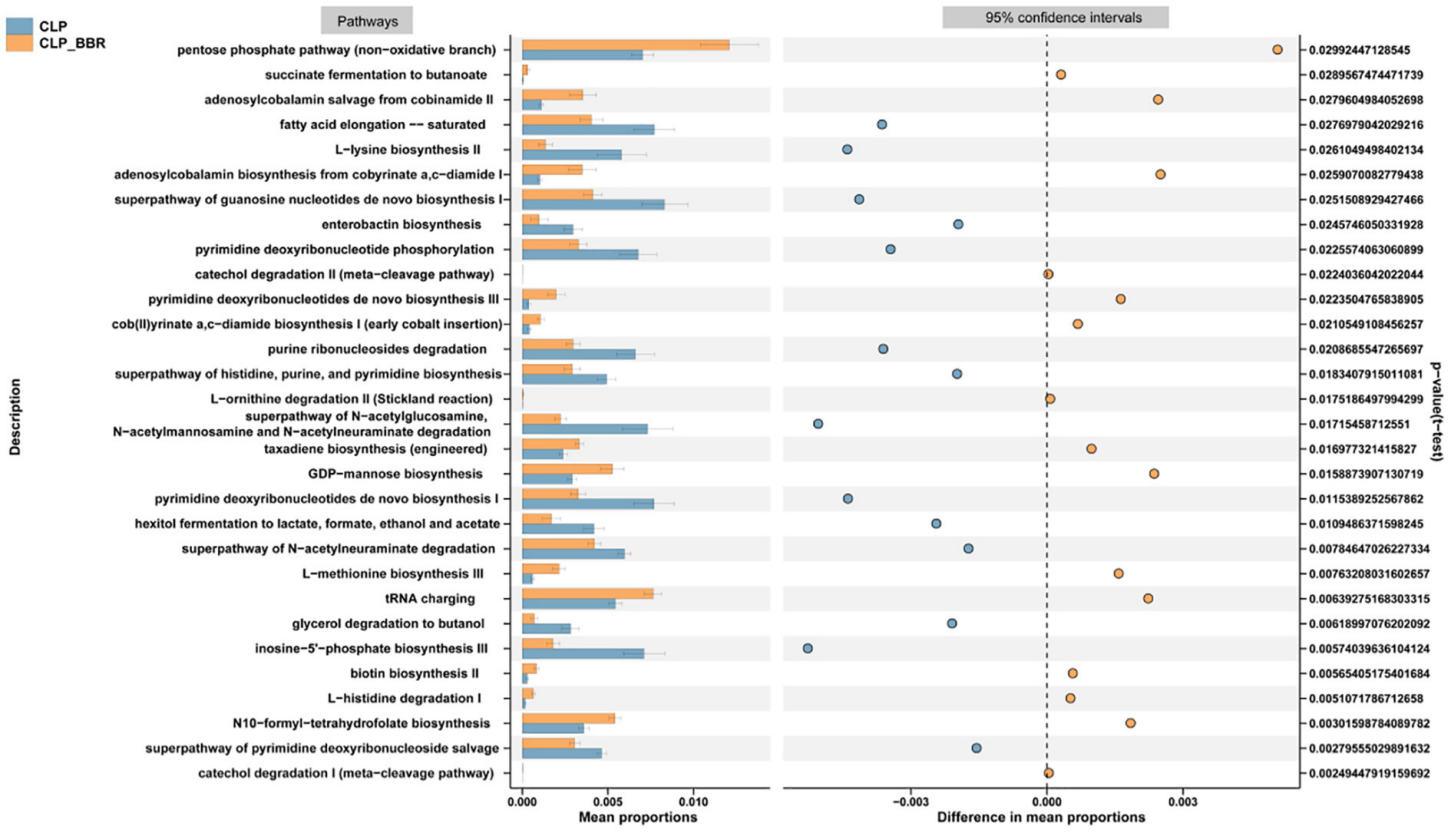
The functional prediction results of PICRUST2. The signaling pathways were forecast based on the species with significant differences in abundance between the CLP and CLP + BBR group and analyzed by *t*-test (*p*-value < 0.05). CLP, cecal ligature and puncture; BBR, berberine.

The mean proportions of the functional prediction results of PICRUST2 showed that the pentose phosphate pathway, the succinate fermentation to butanoate, the adenosylcobalamin salvage from cobinamide II, the adenosylcobalamin biosynthesis from cobyrinate a, c-diamide I, the pyrimidine deoxyribonucleotides *de novo* biosynthesis III, the cob(II)yrinate a, c–diamide biosynthesis I, the taxadiene biosynthesis (engineered), the GDP–mannose biosynthesis, the L-methionine biosynthesis III, the tRNA charging, the biotin biosynthesis II, the L–histidine degradation I, and N10–formyl–tetrahydrofolate biosynthesis pathways were higher in the CLP + BBR group than those in the CLP group (Figure 5). Further, the mean proportions of the L-lysine biosynthesis II, the Enterobactin biosynthesis, the purine ribonucleosides degradation, the super pathway of histidine, purine, and pyrimidine biosynthesis, the pyrimidine deoxyribonucleotides *de novo* biosynthesis I, the hexitol fermentation to lactate, formate, ethanol, and acetate, the super pathway of *N*-acetylneuraminate degradation, the glycerol degradation to butanol, inosine-5'-phosphate biosynthesis III, and the super pathway of pyrimidine deoxyribonucleoside salvage pathways were lower in the CLP + BBR group than those in the CLP group (Figure 5).

### 3.5. The effect of BBR treatment on metabolomic changes in the plasma of CLP rats

**Figure 6:**
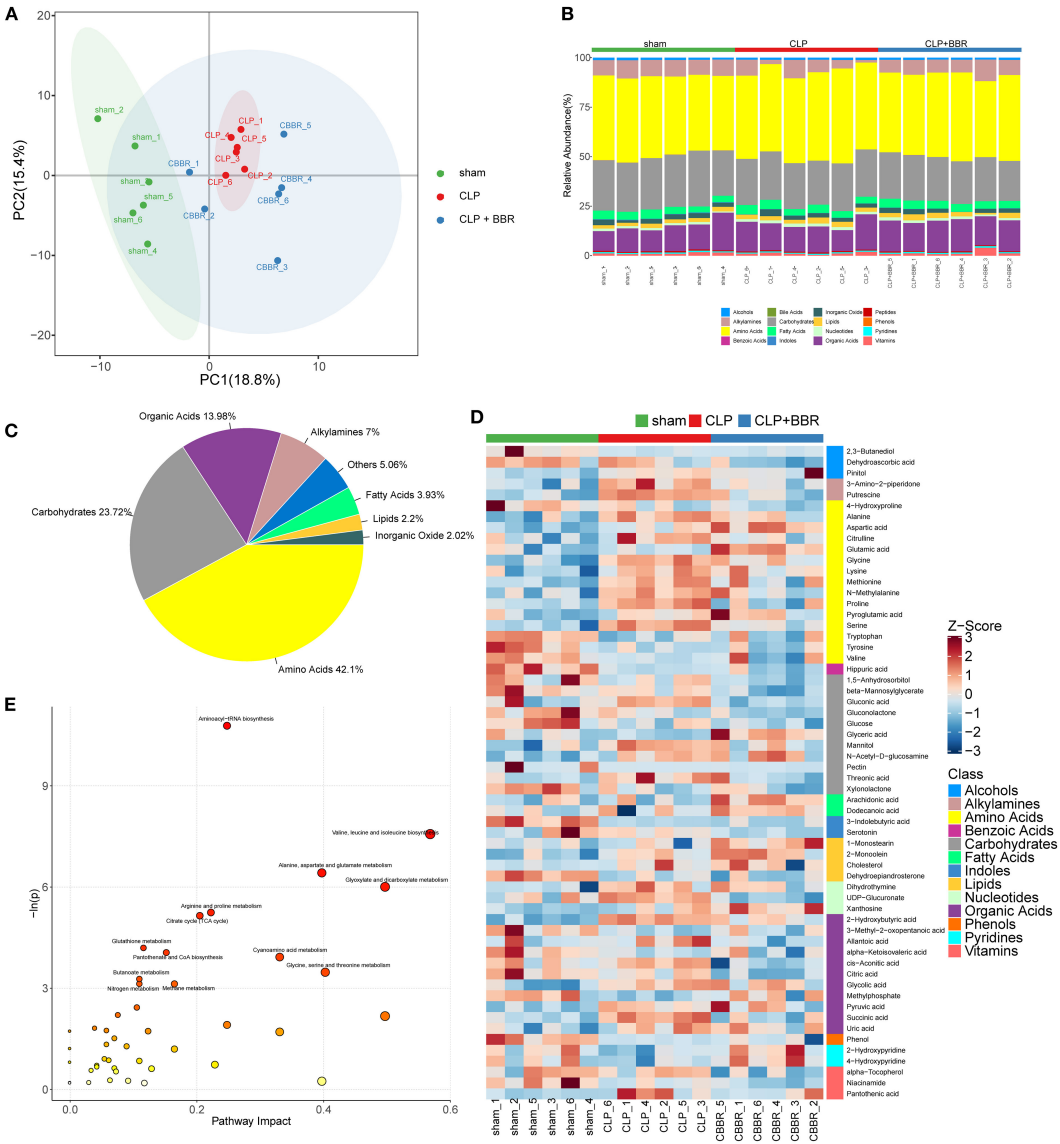
Metabolomic analysis of cecal ligature and puncture (CLP) rats with berberine (BBR) treatment. **(A)** The principal component analysis (PCA) score plot with samples of the sham group, CLP group, and CLP + BBR group. **(B)** The stacked histogram of relative abundance of metabolite class in each sample. **(C)** The proportion of key metabolites is shown in a pie chart; **(D)** The heatmap of potential biomarkers with *p*-value < 0.05 and |log_2_ (fold change)| ≥ 0. In right is the class and each class internal metabolite was ranked by the *Z*-score change feature. **(E)** The bubble plot of pathway enrichment analysis. Each circle corresponds to a metabolic pathway and the abscissa represents “pathway impact.” The bigger circle, the value of “pathway impact.” The color that changes from yellow to red is positively correlated with the negative log *p*-value of the pathway's altered *p*-value.

**Figure 7:**
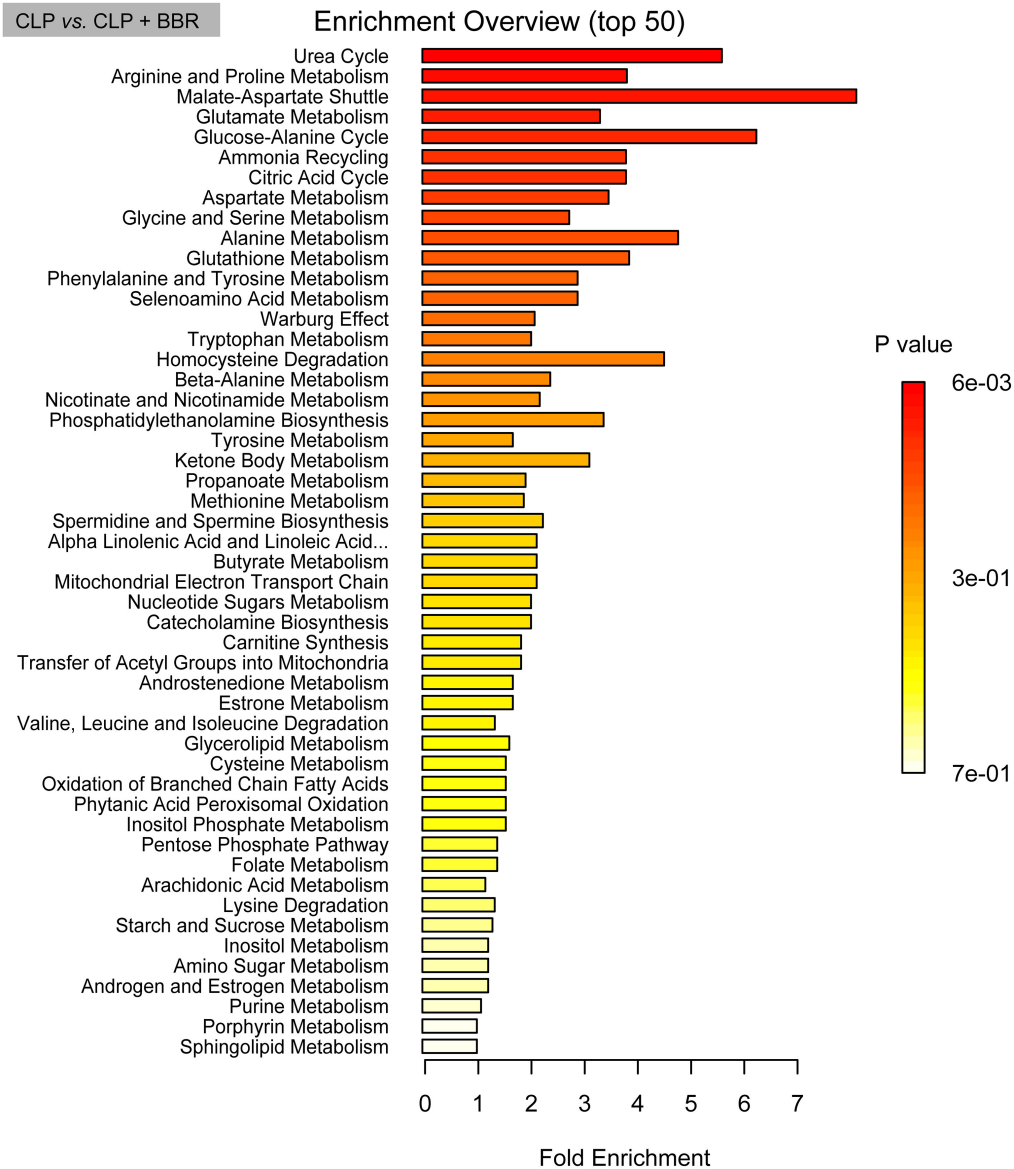
The bar chart of pathway enrichment analysis based on pathway-associated metabolite sets database. The enrichment analysis was based on the metabolites with significant differences between the CLP and CLP + BBR group and analyzed by *t*-test (*p*-value < 0.05). CLP, cecal ligature and puncture; BBR, berberine.

The principal component analysis (PCA) scores of the three groups are shown in Figure 6A, which indicated that the intra-group repeatability of each group was good and that the composition of the CLP + BBR group was closer to that of the sham group than that of the CLP group. A total of 159 metabolites were obtained. They are in 12 classes (Figure 6B), and most of the metabolites belong to amino acids (42.1%), carbohydrates (23.72%), and organic acids (13.98; Figure 6C). Moreover, 60 potential biomarkers among 159 metabolites are shown in Figure 6D. Additionally, the pathway enrichment analysis of potential biomarkers showed that 13 pathways were significantly related, including the aminoacyl-tRNA biosynthesis, the valine, leucine, and isoleucine biosynthesis, the alanine, the aspartate and glutamate metabolism, the glyoxylate and dicarboxylate metabolism, the arginine and proline metabolism, the citrate cycle (TCA cycle), the glutathione metabolism, the pantothenate and CoA biosynthesis, the cyanoamino acid metabolism, the glycine, the serine and threonine metabolism, the butanoate metabolism, the nitrogen metabolism, and the methane metabolism pathways (Figure 6E). The enrichment analysis of SMPDB was based on the metabolites with significant differences between the CLP and CLP + BBR groups. The urea cycle, arginine and proline metabolism, malate-aspartate shuttle, glutamate metabolism, glucose-alanine cycle, and other metabolic pathways were the ones with significant differences (Figure 7).

The aminoacyl-tRNA biosynthesis, the glyoxylate and dicarboxylate metabolism, the alanine, aspartate, and glutamate metabolisms, and the glutathione metabolism pathways were identified as potentially regulated pathways in the comparison of CLP + BBR with sham groups and the CLP group, respectively. In the aminoacyl-tRNA biosynthesis pathway, alanine and serine had relatively high expression in the CLP group compared to the sham group and were decreased in the CLP + BBR group compared to the sham group. Further, compared with the sham group, glutamic acid had an opposite expression trend in the CLP and CLP + BBR groups (Supplementary Figures 1, 2).

### 3.6. The Person correlation analysis between metabolites and intestinal flora

**Figure 8:**
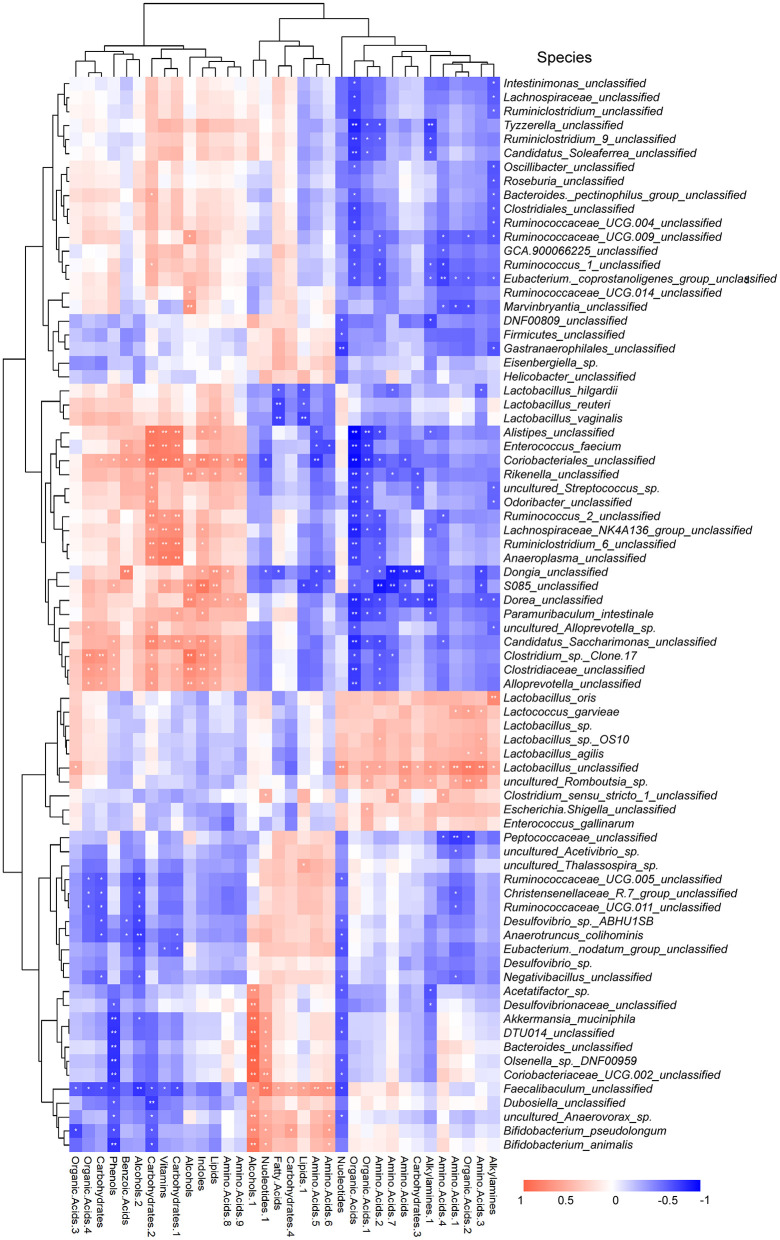
Heatmap for correlation analysis of gut microbiota and serum metabolites. Red (blue) represents positive (negative) correlation, and the whiter the color, the weaker the correlation. The *p < 0.05 and **p < 0.01 indicates that the correlation analysis is statistically significant.

In addition, according to the Person correlation analysis of gut microbiota and metabolomics, it was found that the abundance of microbiota was significantly positively or negatively correlated with the metabolites (Figure 8). For example, *coriobacteriales* were significantly positively correlated with carbohydrates, phenols, benzoic acids, alcohols, vitamins, carbohydrates, indoles, lipids, and amino acids. The *Lactobacillus_unclassified* were significantly positively correlated with alkylamines, amino acids, organic acids, alkylamines, and carbohydrates. *Lactobacillus_agilis* were significantly positively correlated with amino acids and organic acids. *Lactobacillus_sp._OS10* were significantly positively correlated with amino acid 3 and amino acid 2. *Lactobacillus_oris* were significantly positively correlated with alkylamines. *Lactobacillus_vaginalis, Lactobacillus_reuteri, and Lactobacillus_hilgardii* significantly negatively correlated with fatty acids and lipids 1. Among them, *Lactobacillus_hilgardii* was also significantly negatively correlated with amino acids 3 and 7.

## 4. Discussion

According to an analysis of a global disease research report from 1990–2017, the mortality rate of sepsis has been decreasing in recent years. In 2017, however, sepsis accounted for 16.5% of all deaths, constituting a severe burden on human health (30, 31). In addition, in patients with sepsis, the balance of proinflammatory and anti-inflammatory factors determines an individual's outcome (32). Thus, studying effective treatments and drugs for regulating inflammation in patients with sepsis is necessary. Multiple histological injuries, including the lungs, the kidneys, and the spleen, have been observed in rats with sepsis, as well as an increase in a significant number of inflammatory markers, according to studies (33–35). This study's observation of histological damage and inflammatory indexes proved that the CLP rat model was also successfully constructed.

Besides, this study demonstrated that BBR helped heal the histological damage in the lungs, the kidneys, and the ileum of CLP rats and inhibited the levels of IL-1β, IL-6, IL-17A, and MCP-1 in these rats. This demonstrated that BBR treatment could attenuate the multiorgan injury and inflammatory response in rats with sepsis. Similarly, BBR has been proven to inhibit inflammatory cytokines like IL-1β and IL-4 (36). The ameliorative effect of BBR in various animal models of sepsis has been demonstrated (13, 37, 38). BBR is the main active ingredient extracted from medicinal herbs with anti-inflammatory and immunomodulatory biological activities and has the potential to antagonize inflammation and histological damage in patients with sepsis (12). BBR. In addition, this study found that the impaired gut barrier function of CLP rats could be improved with BBR treatment. It has been proven that gut microbiota plays an important role in damaging gut barrier function and causing blood infections (16, 39). Gong's team reported that BBR reduced intestinal mucosal barrier dysfunction (40). Therefore, to reveal the biological mechanism of BBR in improving sepsis, this study further studied the gut microbiota and blood metabolomics of CLP rats.

In this study, 16S rDNA sequencing analysis showed that the abundance of the gut microbiota in the CLP rats was different from that of the sham rats, while after BBR treatment, the abundance of the gut microbiota in the CLP rats was closer to that of the sham rats. A study reported an increased risk of sepsis within 90 days after discharge in patients exposed to high-risk antibiotics during hospitalization (41). High-risk antibiotics implies a high risk of *Clostridioides difficile* infection (CDI). CDI is clinically considered a marker of gut microbiota disruption. In this study, the *Clostridiales* (unclassified) were significantly different between the three groups and were related to the organic acids. Wang's team discovered that the mixture of organic and medium-chain fatty acids could prevent inflammation and gut barrier dysfunction in enterohemorrhagic *Escherichia coli*-infected mice (42).

This study found that nearly 38% of the gut microbiota was significantly negatively correlated with organic acids, including *Clostridiales* (unclassified). The effect of BBR on the bacteria that produce short-chain fatty acids has been proven by Wang's team (36). Besides, in the rat model with 5-fluorouracil-induced intestinal mucositis, BBR notably increased butyrate levels and glutamine levels in feces (43). It is suggested that BBR helped heal histological damage, inflammation, and intestinal permeability in the CLP rats by regulating the organic acids in the gut microbiota, which merits further study and analysis.

In this study, at the genus level, *Muribaculaceae* was the gut microbiota, with a negative correlation with *Lactobacillus*. Further analysis of its expression at the species level found that the *Lactobacillus_crispatus* was significantly increased in CLP rats compared to the sham rats. On the contrary, the *Lactobacillus_unclassified, Lactobacillus_hilgardii, Lactobacillus_reuteri, and Lactobacillus_vaginalis* were significantly decreased in the CLP + BBR group. A study reported that *Bifidobacterium* animalis and *Lactobacillus crispatus* would promote Caenorhabditis Elegans expansion in ICU patients, revealing a significant negative impact of these microbes on host viability and developmental homeostasis (44). In contrast, *Lactobacillu*s*_reuteri* is believed to be able to antagonize neonatal sepsis (45). It is controversial whether *Lactobacillus* is beneficial for human anti-infection (46). It is still worth exploring further which specific level of *Lactobacillus* is crucial for BBR to play an anti-infection role.

Additionally, a study reported the negative relevance of *Muribaculaceae* to IL-6 in the CLP rat brain (29). In this study, *Muribaculaceae* was increased in the CLP + BBR group compared to the CLP group. Furthermore, *Akkermansia muciniphila* was increased by BBR treatment. *Akkermansia_muciniphila* are beneficial to gut commensals that can improve the urinary metabolomic profiles of their hosts (47, 48). BBR was shown to have the potential as a prebiotic to improve *the Akkermansia_muciniphila* growth environment by promoting its abundance (49). Additionally, *Escherichia-Shigella* is a pathogenic bacterium associated with intestinal inflammation that was decreased in the CLP rats with BBR treatment. This may be related to the increase of probiotics such as *Bifidobacterium_pseudolongum*. Pang et al. discovered that *Bifidobacterium animalis* could improve intestinal development and decrease the abundance of *Helicobacter pylori* and *Escherichia-Shigella* in the ileal mucosa of weaning piglets (50). *Muribaculaceae, Akkermansia_muciniphila, and Bifidobacterium_pseudolongum* might be related to *BBR* improving sepsis.

In addition, this study observed that the BBR could improve the proportion of CD4^+^ and CD8^+^ T cells in splenocytes. Studies have reported that CD8^+^ T cells undergo apoptosis and have impaired polyfunctionality in patients with sepsis (51, 52). In particular, it was well established that CD4^+^ and CD 8^+^ T cells were lost by apoptosis during the first week following CLP (53). Additionally, Li et al. (54) reported that the Th17 cells increased in sepsis and the activation of the IL-17-related pathway promotes pyroptosis in pneumonia-induced sepsis. Interestingly, a study about HIV-1-infected patients found the abundance of the gut microbiota correlated with activated CD4^+^ and CD8^+^ T cells (55). Scientists demonstrated the necessity of gut microbiota for Th17 cell differentiation in germ-free mice (56). Th17 cells play a key role in the development of autoimmune disease by producing the proinflammatory cytokines IL-17A (57). Those studies suggest that BBR may regulate the levels of CD4^+^ T cells, CD8^+^ T cells, and CD4^+^ CD25^+^ Foxp3^+^ T cells by improving gut microbiota disorders and decreasing Th17 cells. In this study, a disturbance of the gut microbiota may be one of the reasons for the decreased levels of IL-17A and Th17 cells. Additionally, Tanoue's team proved the immunomodulatory molecule of Treg cells (CD4^+^ CD25^+^ Foxp3^+^ T cells) provided by the gut microbiota (58). Moreover, Ehteshamfar reported that BBR could directly suppress functions and differentiation of proinflammatory Th1 and Th17 cells (12); this suggested the relevance of the gut microbiota on the T cells of CLP rats with BBR treatment. In particular, scientists studied C57BL/6 mice with different microbiota from different dealers and found that the difference in survival rate disappeared after those mice were co-housed (59). It was also found that the gut microbiota was correlated with the survival rate of sepsis. This study found that *Streptococcus_equinus* has a high abundance in the CLP group while having a low abundance in the sham and CLP + BBR groups. A study found the anti-*Streptococcus* antibodies could induce T cells to transmigrate into cardiac tissue to induce inflammation (60). Sikder with co-workers found that β-hemolytic group C streptococci and group G streptococci caused IL-17A/interferon γ-induced myocarditis and valvulitis (61). Modern research argues that the gut microbiota can modulate immunity by altering metabolite levels within the host (62, 63). The influence of gut microbiota abundance changes may be one of the key research directions to improve immunity against sepsis in the future.

This study predicted that the biological mechanism of BBR to ameliorate sepsis involves metabolic pathways, including glycolysis, the nucleotide-related pathways, and the amino acid-related pathways. As Figures 5, 6E show, the glycogen metabolism pathway is the relevant pathway of pertinent flora upregulation and abundance after the action of BBR, and it is also the metabolite enrichment pathway with statistical significance, suggesting that the glycogen metabolism pathway was imported in BBR improving CLP-rats. Particularly, the pentose phosphate pathway (PPP) was potentially relevant in BBR improving CLP-rats according to the data of 16S rDNA sequencing and metabolomics analysis. A study reported that glycogen metabolism regulates inflammatory responses in patients with sepsis (64). Additionally, PPP was a key pathway for regulating Treg cells (65). Daneshmandi et al. (66) reported that blocking 6-phosphogluconate dehydrogenase in the oxidative PPP resulted in a substantial reduction of Tregs' suppressive function and shifts toward Th1, Th2, and Th17 phenotypes, which led to the development of the fetal inflammatory disorder in a mouse model. It suggested that BBR may inhibit Treg cells by blocking PPP in Tregs, which warrants further study. Additionally, the metabolites were mainly involved in the urea cycle of CLP-rats after BBR treatment. A study reported that CLP results in altered renal metabolism (67). In this study, BBR might ameliorate the renal injury present in CLP rats by regulating the gut microbiota, which may be associated with a large alteration of urea cycle metabolites.

Additionally, the aminoacyl-tRNA biosynthesis pathway was potentially regulated in the comparison of CLP + BBR with the sham group and the CLP group, respectively. A study reported that the lungs of pigs with sepsis induced by *Pseudomonas aeruginosa* had significant changes in aminoacyl-tRNA biosynthesis (68). In this pathway, this study found that serine had relatively high expression in the CLP group compared to the sham group and was decreased in the CLP + BBR group compared to the sham group. A study reported that serine metabolism was related to IL-1β mRNA expression (69). These suggested serines may play a key role in regulating the aminoacyl-tRNA biosynthesis pathway to increase the IL-1β level.

Additionally, glutamic acid was a potential metabolic biomarker with the largest number of pathways, suggesting that glutamic acid-related metabolism may be involved in the development of sepsis. Another study reported that the amino acid ratio is significantly correlated with elevated IL-6 levels (70). In conclusion, this study clearly shows that BBR protects rats from sepsis, which is significantly related to changes in gut microbiota and metabolites.

Although we could not determine whether the gut microbiota is a decisive factor in the improvement of sepsis by BBR, this study still provided an experimental basis for the treatment of sepsis and provided a new perspective and scientific basis for the study of the biological mechanism by which BBR improves sepsis.

## 5. Conclusion

This study proved the protective effect of BBR on the CLP rats in terms of histological damage to the lungs, the kidneys, and the ileum and the levels of IL-1βIL-6, IL-17A, MCP-1, DAO, and FD-40. By using 16S rDNA and metabolomics techniques, we proved the effects of BBR on gut microbiota and plasma metabolites. It was demonstrated that BBR improved CLP rats by restoring gut microbiota abundance and changing plasma metabolites. This study provided a scientific basis for BBR to improve sepsis and a new direction for the study of the biological mechanism.

## Data availability statement

The raw data supporting the conclusions of this article will be made available by the authors, without undue reservation.

## Ethics statement

The animal study was reviewed and approved by Animal Experimentation Ethics Committee of Zhejiang Eyong Pharmaceutical Research and Development Center [SYXK (Zhe) 2021-0033].

## Author contributions

Conception and design of the research by HP, LH, and YB. Acquisition of data by WS and ZD. Analysis and interpretation of data by WS and CJ. Statistical analysis by HP and WS. Obtaining funding by LH and WS. Drafting the manuscript by HP and CJ. Revision of manuscript for important intellectual content by HP and JZ. All authors contributed to the article and approved the submitted version.
