# Supplementary material for: Study on the protective effect of berberine treatment on sepsis based on gut microbiota and metabolomic analysis
Source: Front Nutr. 2022 Dec 19;9:1049106. doi: 10.3389/fnut.2022.1049106 (PMC9806126; doi:10.3389/fnut.2022.1049106)
Supplement: Supplementary file 1 [file Data_Sheet_1.docx]

**Supplement figures**

**
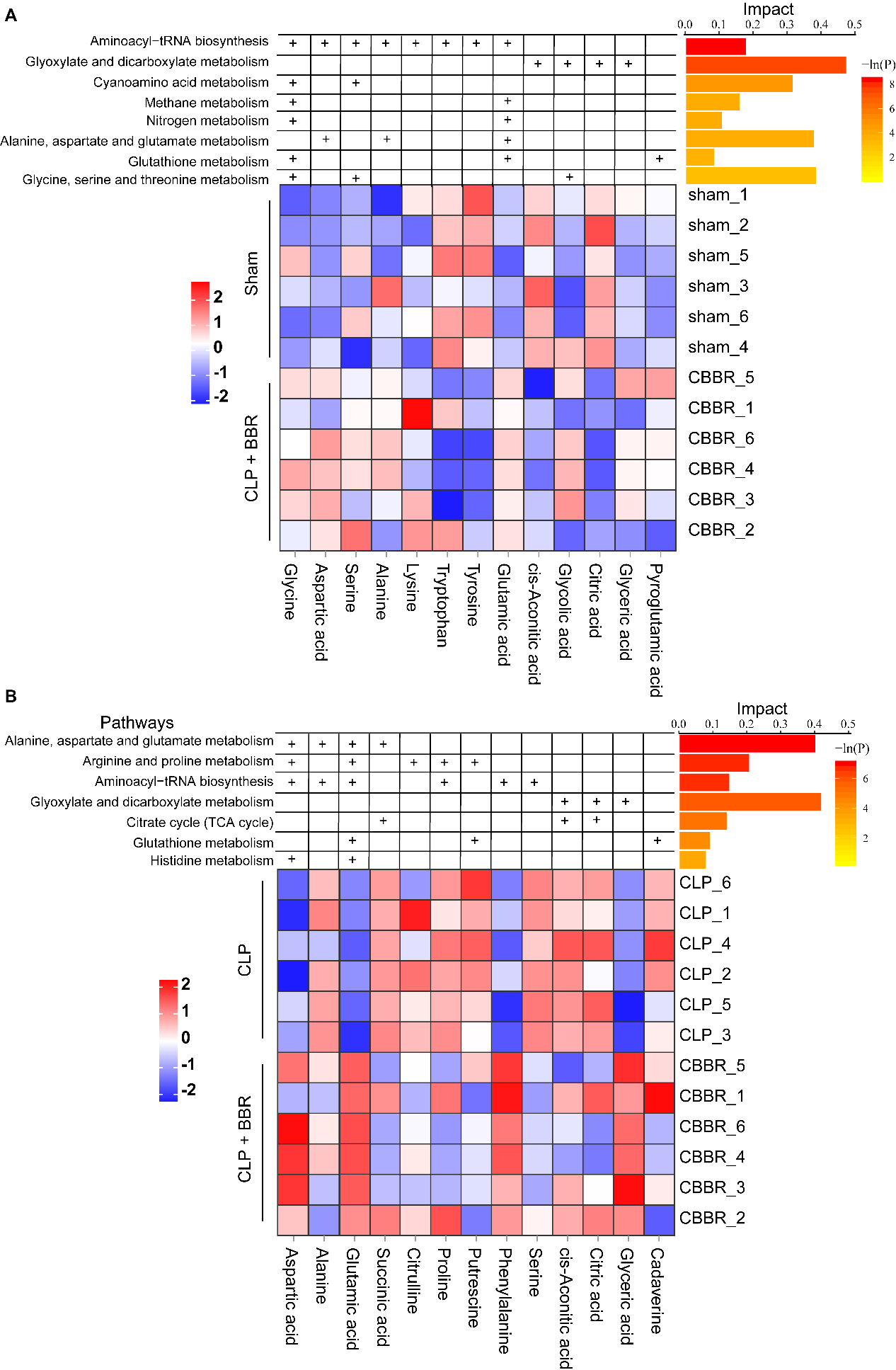
**

**Supplementary figure 1** The potential biomarkers screened by Mann-Whitney U Test (*P* < 0.05, |log_2_(fold change) | ≥ 0). Then potential biomarkers were enrichment analysis by rno database. (A) The bar chart was shown the pathways with *p* < 0.05 and the heat map was shown the related potential markers, that was in the sham group compared to the CLP + BBR group. (B) The bar chart was shown the pathways with *p* < 0.05 and the heat map was shown the related potential markers, that was in the CLP group compared to the CLP + BBR group. The plus signs marked the markers with significant differences that were involved in this signaling pathway. The length of the bar chart on the right represented the enrichment degree of the pathway, the greater the impact value represented the greater the enrichment. And the bar chart color represented the log (*p*-value), and the tendency of color tended to be red indicating a smaller *p*-value. Additionally, in the heat map, blue indicated low expression, and red indicated high expression.


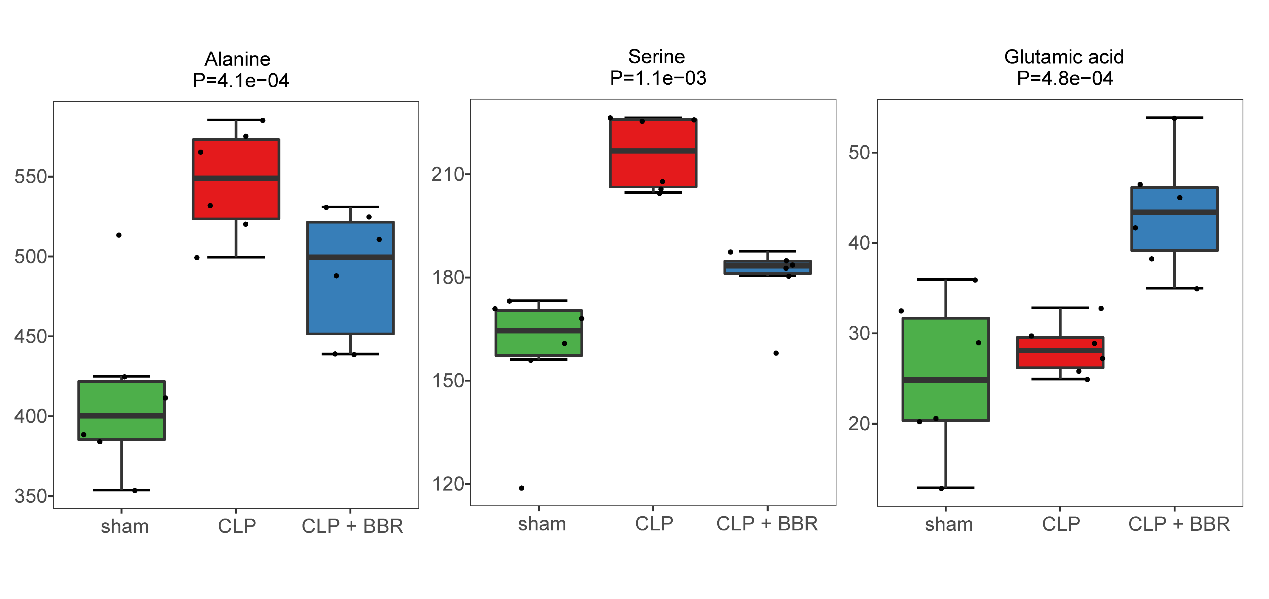


**Supplementary figure 2** The serum metabolite levels of alanine, serine, and glutamic acid in the sham, CLP, and CLP + BBR groups.
